# MiRNA-22 inhibits oncogene galectin-1 in hepatocellular carcinoma

**DOI:** 10.18632/oncotarget.10981

**Published:** 2016-08-01

**Authors:** Yu You, Jia-Xin Tan, Hai-Su Dai, Hao-Wei Chen, Xue-Jun Xu, Ai-Gang Yang, Yu-Jun Zhang, Lian-Hua Bai, Ping Bie

**Affiliations:** ^1^ Department of Hepatobiliary Surgery Institute, South Western Hospital, Third Military Medical University, Chongqing 400038, China

**Keywords:** hepatocellular carcinoma, hepatic stellate cells, galectin-1, miRNA-22

## Abstract

Hepatic stellate cells (HSCs) induce immune privilege and promote hepatocellular carcinoma (HCC) by suppressing the immune system. On the other hand, galectin-1 and miRNA-22 (miR-22) are dysregulated in HCC and serve as prognostic indicators for patients. In this study, therefore, we measured galectin-1 and miR-22 expression in HSCs isolated from HCC tissues (Ca-HSCs), and in normal liver tissues (N-HSCs) as a control. We also investigated the apoptosis rate among T cells and the production of cytokines (IFN-η and IL-10) in HSCs co-cultured with T cells. And we used immunohistochemical staining to tested for correlation between galectin-1 expression, CD3 expression and clinicopathological features in 162 HCC patients. Our results showed that galectin-1 expression was much higher in Ca-HSCs than in N-HSCs. Overexpression of galectin-1 promoted HSC-induced T cell apoptosis and cytokine production (IFN-η and IL-10), while miR-22 expression inhibited it. Galectin-1 expression correlated negatively with miR-22 expression in HSCs. High galectin-1 and low CD3 expression levels were associated with poor prognosis in HCC patients. These results suggest that the immunosuppressive microenvironment promoted by HSC-derived galectin-1 in HCC can be inhibited by miR-22. Galectin-1 and miR-22 could potentially serve as prognostic markers and therapeutic targets in HCC.

## INTRODUCTION

Hepatocellular carcinoma (HCC) is the most common type of primary tumour in the liver and is one of the most common human cancers worldwide [1]. In spite of major advances in understanding liver carcinogenesis, HCC remains deadly; therefore, innovative therapies are needed to improve prognosis for patients [2]. The tumour cell microenvironment plays a key role in cancer pathogenesis [2] and new therapeutic strategies that target it appear to be promising. In the liver, hepatic stellate cells (HSCs) create an immunosuppressive environment that promotes HCC growth [1–3]. Furthermore, apoptosis induction in T cells also promotes HCC [4, 5].

Galectin-1, a member of the galectin family of β-galactoside-binding proteins, is a homodimer of 14 kDa subunits that possess two β-galactoside binding sites [6]. Galectin-1 modulates cell proliferation, adhesion, and migration, all of which are essential for tumorigenesis [7–9], and also regulates the interaction between tumour cells and components of the tumour microenvironment [10, 11]. Furthermore, galectin-1 levels can be used as a marker to predict poor prognosis of many types of cancers, including HCC [12, 13]. While galectin-1 is expressed in human HuH-7 cells, JHH-6 cells and tumour hepatocytes of human HCC tissue sections [14], galectin-1 levels in hepatocytes are significantly lower in HCC specimens than in normal liver and cirrhotic specimens [14, 15]. Moreover, galectin-1 expression is lower in neoplastic hepatocytes of HCC tumours than in surrounding stromal tissue. On the other hand, galectin-1 levels in stromal cells are higher in HCC specimens than in normal liver and cirrhotic specimens [14, 15]. Moreover, endogenous galectin-1 plays a key role in tumour immune privilege due to its capacity to induce apoptosis in activated T cells [7], which is relatively similar to the immunomodulatory properties of HSCs. Indeed, galectin-1 expression has been detected in rat HSCs [16, 17]. Therefore, we investigated whether human HSCs express galectin-1 and whether galectin-1 contributes to HSC-mediated immunomodulatory functions and tumour immune privilege in HCC.

We also aimed to explore the upstream signalling pathway of galectin-1. MicroRNAs (miRNAs) are an abundant class of small noncoding RNAs that are 20 to 25 nucleotides in length, which participate in diverse biological processes, including cancer development [18]. Recently, miR-22 has been shown to be downregulated in many types of cancers, including HCC, resulting in an anti-proliferative effect [19]. Moreover, target prediction analysis identified galectin-1 as a predicted target of miR-22. Thus, in this study, we aimed to determine whether miR-22 is involved in the immunomodulatory functions of galectin-1 and HSCs.

## RESULTS

### Activated human HSCs can induce T cell apoptosis

**Figure 1:**
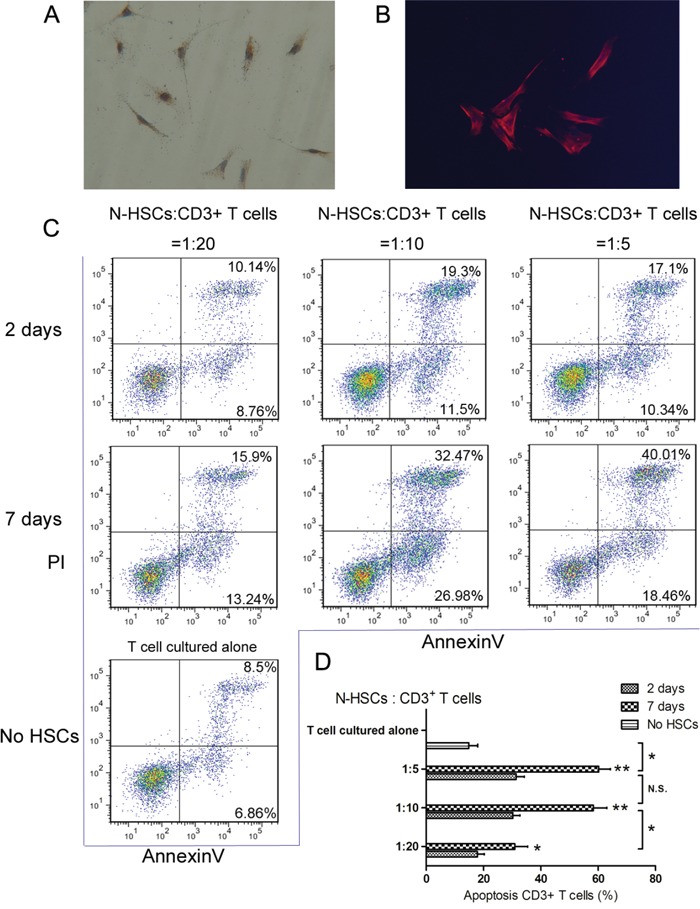
Activated α-SMA-positive HSCs could induce CD3+ T cells apoptosis Primary N-HSCs (HSCs isolated from normal liver tissues) were analysed for **A-B.** α-SMA expression by immunofluorescence and immunohistochemical assays and for **C-D.** the ability to induce apoptosis in T cells by co-culture assays. For the co-culture assays, CD3+ T cells were co-cultured with quiescent HSCs (pre-cultured for 2 days) or activated HSCs (pre-cultured for 7 days) at different ratios (HSC:T = 1:5, 1:10 and 1:20). The data for each group are representative of 4 independent experiments representing 4 different liver samples and repeated 3 times. T cells were cultured alone as a negative control. After the cells were co-cultured for 48 hours, T cell apoptosis was measured by flow cytometry (annexin V-FITC apoptosis detection). All the groups with activated HSCs showed significantly higher apoptosis rates for T cells alone than for groups with quiescent HSCs. Among the groups with activated HSCs, the ratios 1:5 and 1:10 showed the highest T cell apoptosis rate. Data are shown as the means (± SD) of triplicates (n = 4). *P < 0.05, **P < 0.01.

To identify HSCs, expression of α-SMA (a marker of activated HSCs) was confirmed through immunofluorescence and IHC assays of N-HSCs cultured for seven days (Figure 1A and 1B). After seven days in culture, primary HSCs are known to be activated and to differ from quiescent HSCs (after two days in culture) in biological function [20, 21]. HSCs can be immunosuppressive capacity owing to their ability to induce apoptosis in T cells [5]. Thus, to test the pro-apoptotic effect of human HSCs against T cells, CD3+ T cells were co-cultured with N-HSCs (quiescent HSCs or activated HSCs) at different ratios (HSC:T = 1:5, 1:10, and 1:20; T cells cultured alone served as a negative control). As shown in Figure 1C and 1D, activated HSCs at a ratio of 1:10 induced an optimal pro-apoptotic effect on CD3^+^ T cells. Based on this result, HSCs cultured for seven days at a ratio of 1:10 (HSC:T) were used in the following co-culture experiments.

### Galectin-1 is expressed in human activated HSCs

**Figure 2:**
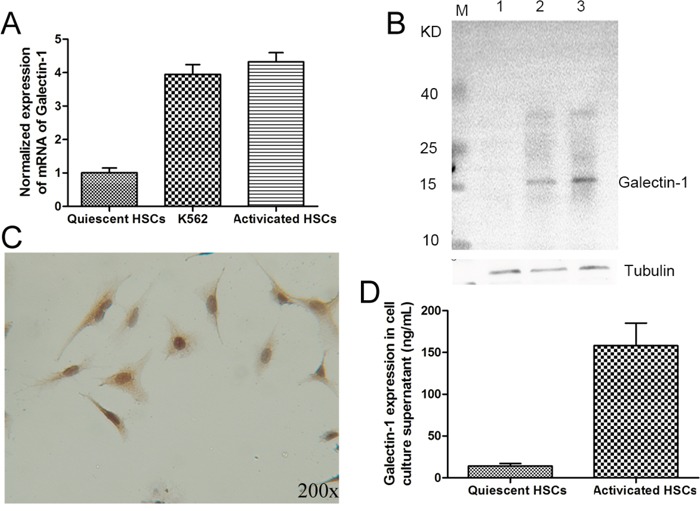
The expression of galectin-1 in human HSCs Primary HSCs isolated from normal liver tissues were tested for galectin-1 expression by **A.** RT-qPCR, **B.** western blot (line 1 for quiescent HSCs, line 2 for K562 cell line and line 3 for activated HSCs, 20μg per lane), **C.** immunohistochemical assays for activated HSCs, and **D.** ELISA (20,000 HSCs per well). In A. and B., the K562 cell line was used as positive control for galectin-1 mRNA and protein expression. Data are shown as the means (± SD) of triplicates (n = 4).

The K562 cell line expresses galectin-1 mRNA and protein [22]; and was thus used as a positive control in the RT-qPCR and WB analyses. As shown in Figure 2A-2C, the RT-qPCR, WB, and IHC results confirmed galectin-1 gene and protein expression in activated HSCs. In fact, the results showed that activated human HSCs expressed galectin-1 mRNA and protein at levels comparable to those of the positive control (Figure 2A and 2B). Moreover, analysis of the cell culture supernatant by ELISA suggested that galectin-1 could be released by activated HSCs (Figure 2D).

### Galectin-1 expression in HSCs contributes to HSC-induced apoptosis of T cells

**Figure 3:**
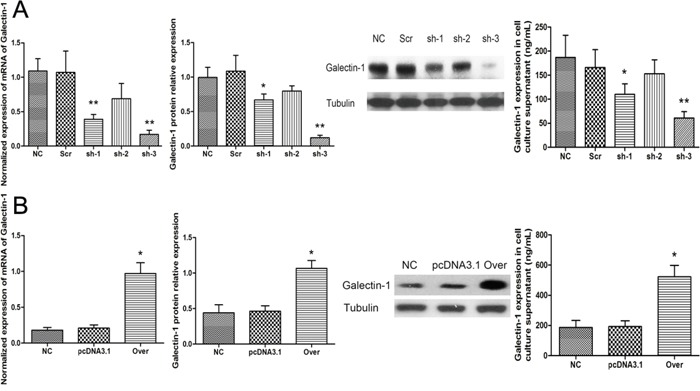
Knockdown and overexpression of galectin-1 in N-HSCs After the cells were transfected, qPCR (n = 3), western blot (30μg per lane, n = 3) and ELISA (20,000 HSCs per well, n = 4) were used to analyse galectin-1 knockdown **A.** and overexpression **B.** in HSCs. Data are shown as the means (± SD) of triplicates. *P < 0.05, **P < 0.01. NC, negative control group; Scr, non-targeting scrambled sequence group; sh, small hairpin RNA sequence transfection group; pcDNA3.1, negative control group; Over, galectin-1 overexpression group.

**Figure 4:**
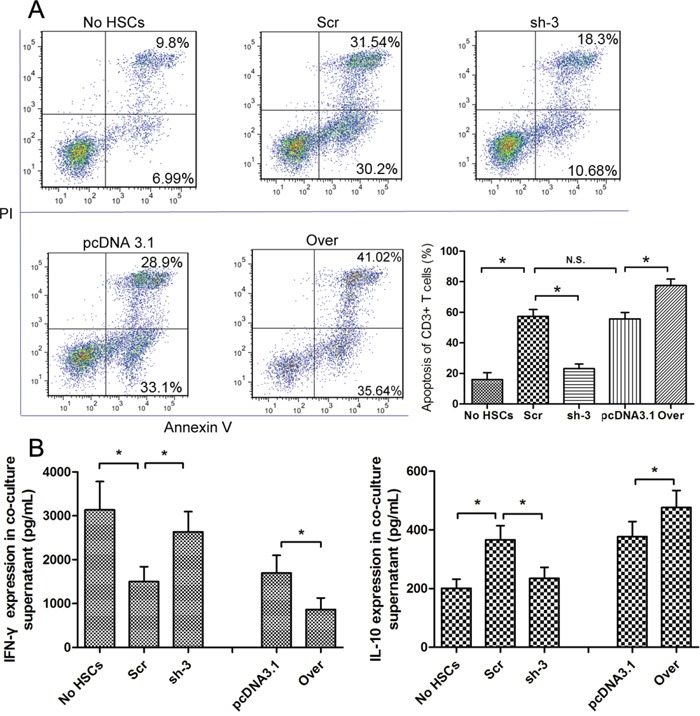
The expression of galectin-1 in HSCs promotes HSC-induced T cell apoptosis and Th1/Th2 cytokine balance skewing **A.** Flow cytometry (annexin V-FITC apoptosis detection) analyses to detect T cell apoptosis in CD3+ T cells, cultured alone or co-cultured with HSCs subjected to different pre-treatments (cell transfection for galectin-1 knockdown and overexpression: sh-3 group versus Scr group; Over group versus pcDNA3.1 group) for 48 hours at a ratio of 10:1 (T:HSC), **B.** ELISA showing the levels of cytokines (IFN-γ and IL-10) in the supernatant. Data are shown as the means (± SD) of triplicates (n = 7). *P < 0.05. NC, negative control group; Scr, non-targeting scrambled sequence group; sh, small hairpin RNA sequence transfection group; pcDNA3.1, negative control group; Over, galectin-1 overexpression group; No HSCs, T cells cultured alone.

To determine whether the pro-apoptotic effect of HSCs on CD3^+^ T cells is involved in the expression of galectin-1, we used RNA interference and gene overexpression to knockdown and overexpress galectin-1 in HSCs, respectively. Galectin-1 knockdown and overexpression in N-HSCs were confirmed by qPCR, WB, and ELISA analyses (Figure 3). According to these results, the sh-3 group versus the Scr group (Figure 3A) and the Over group (galectin-1-overexpression group) versus the pcDNA3.1 group (Figure 3B) were selected (for galectin-1 knockdown and overexpression, respectively) for subsequent experiments to explore the role of galectin-1 in HSCs. As shown in Figure 4A, in the co-culture experiment (N-HSCs and CD3^+^ T cells), galectin-1 knockdown significantly suppressed the pro-apoptotic effect of the N-HSCs, whereas galectin-1 overexpression markedly increased the apoptosis of CD3^+^ T cells, suggesting that galectin-1 promotes apoptosis in HSCs.

### Galectin-1 is involved in HSC-induced Th1/Th2 cytokine balance skewing

Changes in the local immune environment can promote cancer development [2]. Apart from T cell apoptosis, we also examined the Th1/Th2 cytokine balance status to determine the effects of HSCs on the local immune environment. The expression of IFN-γ (as a Th1 cytokine) and IL-10 (as a Th2 cytokine) in the co-culture supernatant was examined by ELISA. As shown in Figure 4B, N-HSCs could decrease IFN-γ expression and increase IL-10 expression, indicating that HSCs could skew the Th cytokine balance from Th1 to Th2. Moreover, this effect could be enhanced or suppressed by galectin-1 overexpression and knockdown, respectively (Figure 4B). Taken together, the above-described results show that HSC-derived galectin-1 contributes to HSC-induced T cell apoptosis and Th1/Th2 cytokine balance skewing, influencing the immunosuppressive capacity of HSCs.

### HSCs from HCC tissues exhibit higher galectin-1 expression and more powerful immunosuppressive capacity than HSCs from normal liver samples

**Figure 5:**
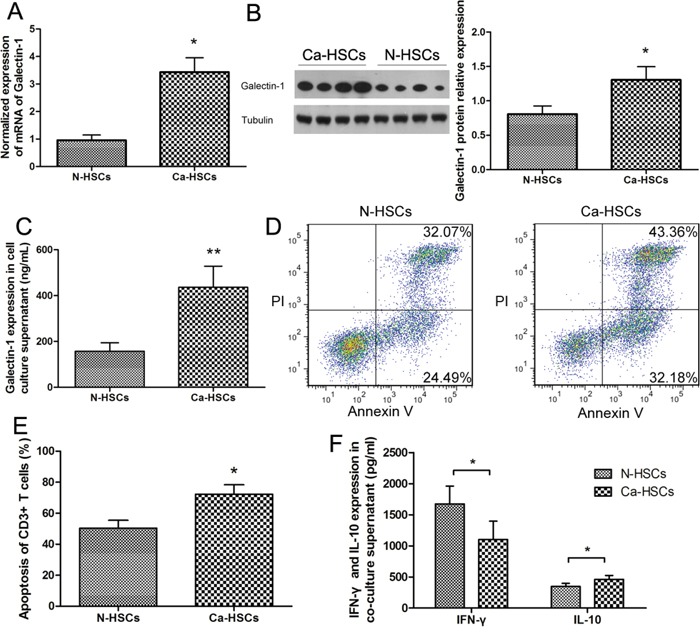
Ca-HSCs showed a higher galectin-1 expression and a stronger immunosuppressive capacity than N-HSCs Expression of galectin-1 by **A.** RT-qPCR, **B.** western blot (30μg per lane), and **C.** ELISA (20,000 HSCs per well) (n = 4) in primary HSCs isolated from normal liver tissues (N-HSCs) or HCC tissues (Ca-HSCs). **D-E.** T cell apoptosis and ELISA analyses of CD3+ T cells co-cultured with N-HSCs (isolated from normal liver samples) or Ca-HSCs (isolated from HCC samples). **F.** Levels of cytokines (IFN-γ and IL-10) in the supernatant (n = 6) measured by flow cytometry (annexin V-FITC apoptosis detection). Data are shown as the means (± SD) of triplicates. *P < 0.05, **P < 0.01.

To determine the contribution of galectin-1 in HSCs to the development of HCC, we compared the expression levels of galectin-1 in N-HSCs and Ca-HSCs (HSCs isolated from HCC tissues) through RT-qPCR, WB, and ELISA analyses. Figure 5A, 5B and 5C show higher expression of galectin-1 in Ca-HSCs than in N-HSCs. We then tested for differences between the immunosuppressive capacity of N-HSCs and Ca-HSCs (Figure 5D and 5E). After co-culture (T cells with N-HSCs or Ca-HSCs), the Ca-HSC group showed a higher T cell apoptosis rate (Figure 5D and 5E) and higher IL-10 expression (Figure 5F), whereas IFN-γ expression showed the opposite trend, as illustrated in Figure 5F. These results demonstrate the higher galectin-1 expression and stronger immunosuppressive capacity of Ca-HSCs compared to those of N-HSCs.

### MiRNA-22 is involved in the expression of galectin-1 and the immunomodulatory capacity of HSCs

**Figure 6:**
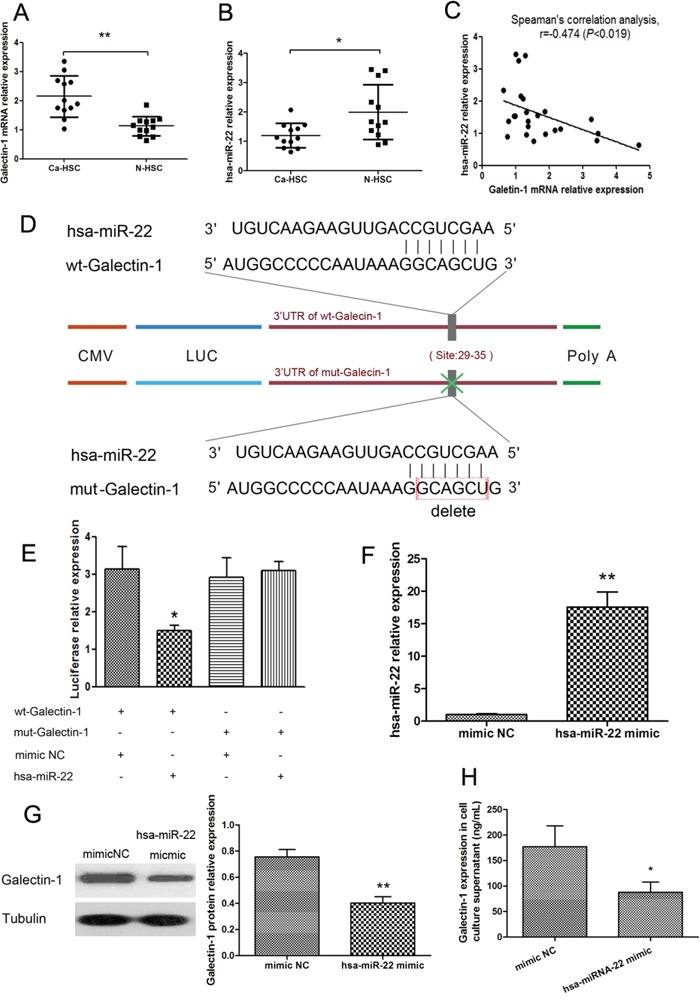
Negative regulation relationship between galectin-1 and miR-22 expression in HSCs Expression of **A.** galectin-1 mRNA, **B.** miR-22 expression, and **C.** their relationship, in N-HSCs (n = 12) and Ca-HSCs (n = 12) measured by qPCR. **D.** Genetic constructs: 3′-UTR of galectin-1 cloned downstream of a luciferase reporter gene (wt-galectin-1), and its mutant version (mut-galectin-1). **E.** Luciferase activity detected by a dual-luciferase reporter assay in HSCs co-transfected for 48 hours with wt-galectin-1/mut-galectin-1 vector and miR-22 expression plasmid/negative control (mimic NC; n = 3 for each group). **F.** MiR-22 expression measured by qPCR in N-HSCs transfected with hsa-miR-22 mimic (n = 4). **G.** Expression of galectin-1 in HSCs measured by western blot (30μg per lane) and **H.** ELISA (20,000 HSCs per well). Data are shown as the means (± SD) of triplicates. *P < 0.05, **P < 0.01. Wt-galectin-1, luciferase reporter gene was cloned with wild type galectin-1; mut-galectin-1, mutant version of galectin-1; mimic NC, mimic negative control; hsa-miR-22, *Homo sapiens* miRNA-22.

MiR-22 has been recently reported to be downregulated in HCC [19]. Furthermore, galectin-1 has been predicted to be a target of miR-22. Therefore, we hypothesized that the decreased expression of miR-22 in HCC might be associated with the increased expression of galectin-1 in HSCs. Analyses of N-HSCs (n=12) and Ca-HSCs (n=12) indicated a significant negative correlation between galectin-1 and miR-22 expression in HSCs (Figure 6A-6C). Additionally, Ca-HSCs showed higher galectin-1 expression and lower miR-22 expression than N-HSCs (Figure 6A and 6B). To determine whether galectin-1 is a direct target of miR-22, we cloned the 3′-UTR of galectin-1 downstream of a luciferase reporter gene (wt-galectin-1); its mutant version (mut-galectin-1) was also constructed. We co-transfected the wt-galectin-1/mut-galectin-1 vector and the miR-22 expression plasmid/mimic NC into HSCs and measured the resulting luciferase activity (Figure 6D and 6E). As shown in Figure 6E, the luciferase activity of miR-22-transfected cells was decreased compared with that of NC cells. Moreover, the miR-22-mediated repression of luciferase activity was abolished by the mutant putative binding site. Taken together, these results indicated a direct negative regulatory relationship between HSC-derived galectin-1 and miR-22 expression. We then transfected the hsa-miR-22 mimic into N-HSCs and then examined miR-22 and galectin-1 expression in HSCs. The results showed that hsa-miR-22 mimic transfection could effectively increase miR-22 expression level in HSCs (Figure 6F) and lead to a marked decrease in galectin-1 protein expression in HSCs overexpressing miR-22 (Figure 6G and 6H).

**Figure 7:**
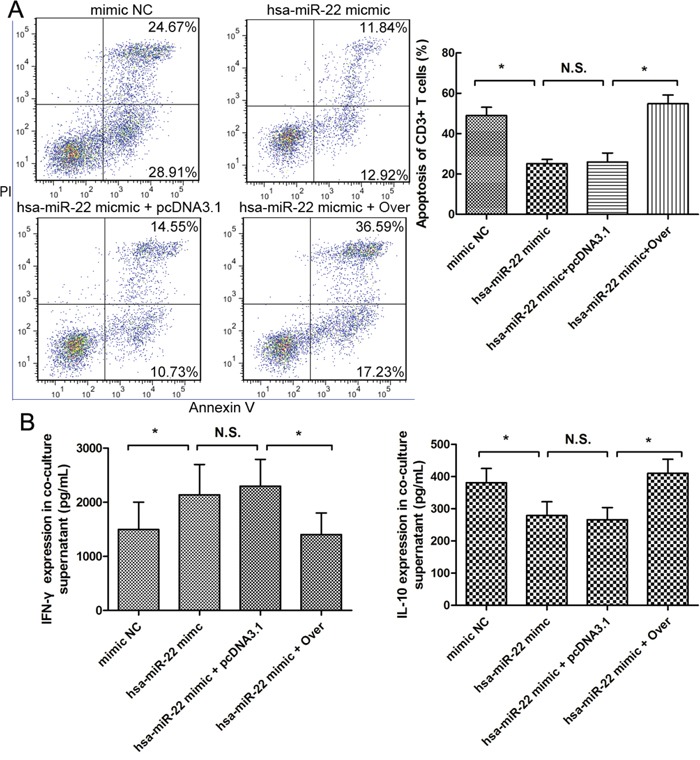
Role of miR-22 in HSC-derived T cell apoptosis and Th cytokine balance skewing T cell apoptosis and cytokine (IFN-γ and IL-10) levels in the supernatant of 4 groups of HSCs isolated from normal liver samples and co-cultured with CD3+ T cells for 48 hours at a ratio of 1:10 (HSC:T) (n = 6 for each group), measured by **A.** flow cytometry and **B.** Elisa. Each of the 4 groups was subjected to different pre-treatments: transfection with 1) negative control mimic for miR-22 (mimic NC), 2) miRNA-22 of homo sapiens type mimic (hsa-miRNA-22 mimic), 3) hsa-miRNA-22 mimic plus galectin-1 overexpression plasmid (hsa-miRNA-22 mimic + Over), and 4) hsa-miRNA-22 mimic plus negative control for galectin-1 overexpression (hsa-miRNA-22 mimic + pcDNA3.1) Data are shown as the means (± SD) of triplicates. N.S. for P > 0.05; *P < 0.05.

We also tested the effects of miR-22 on the immunosuppressive capacity of HSCs. We co-cultured CD3^+^ T cells with N-HSCs subjected to different pre-treatments (miR-22 overexpression or miR-22 overexpression plus galectin-1 overexpression) and then measured HSC-induced T cell apoptosis and Th cytokine balance skewing through flow cytometry (Figure 7A) and ELISA (Figure 7B). The results shown in Figure 7 indicated that miR-22 overexpression could effectively inhibit the pro-apoptotic function and Th1/Th2 cytokine balance skewing of HSCs, and this inhibitory effect could be almost completely inhibited by galectin-1 overexpression. Taken together, the above-described results suggested that miR-22 could inhibit galectin-1 expression in HSCs and its immune effects.

### Galectin-1 expression in HSCs is correlated with CD3 expression and the clinicopathological features of HCC

**Figure 8:**
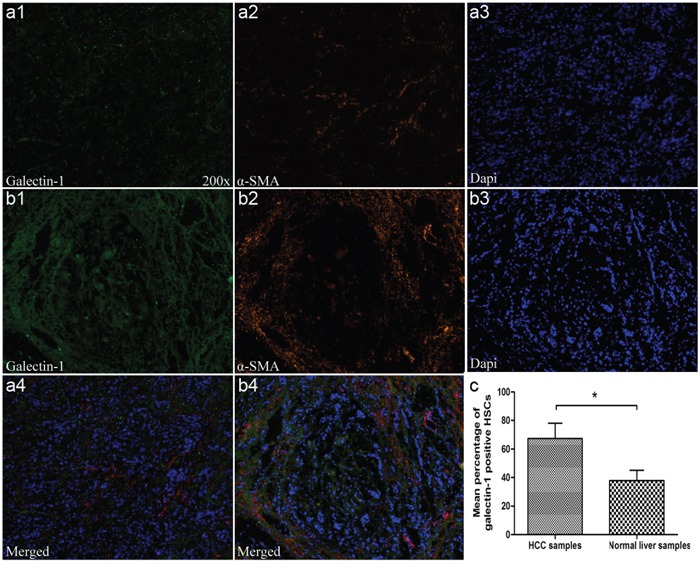
Immunofluorescent labelling for galectin-1 and α-SMA in liver samples **A1-A4.** α-SMA and galectin-1 levels in normal liver samples (n = 12) and **B1-B4.** HCC (n = 31) by immunofluorescence. **C.** Levels of HSC-derived galectin-1 assessed by evaluating the number of galectin-1-positive hepatic stellate cells (both positive for galectin-1 and α-SMA) over the total number of HSCs (only positive for α-SMA). Data are shown as the means (± SD). Magnification: ×100. *P < 0.05.

To verify the expression of HSC-derived galectin-1 in liver samples, we measured the expression of α-SMA and galectin-1 in HCC (n = 31) and normal fresh-frozen liver specimens (n = 12) using immunofluorescence (Figure 8). The levels of HSC-derived galectin-1 were scored by evaluating the number of galectin-1-positive hepatic stellate cells (positive for both galectin-1 and α-SMA) over the total number of HSCs (positive for α-SMA). As shown in Figure 8, galectin-1 was detected in the α-SMA-positive regions, and the expression of HSC-derived galectin-1 was significantly higher in the HCC specimens than in normal liver specimens (Figure 8C), indicating that more HSCs in HCC than in the normal liver express galectin-1.

**Figure 9:**
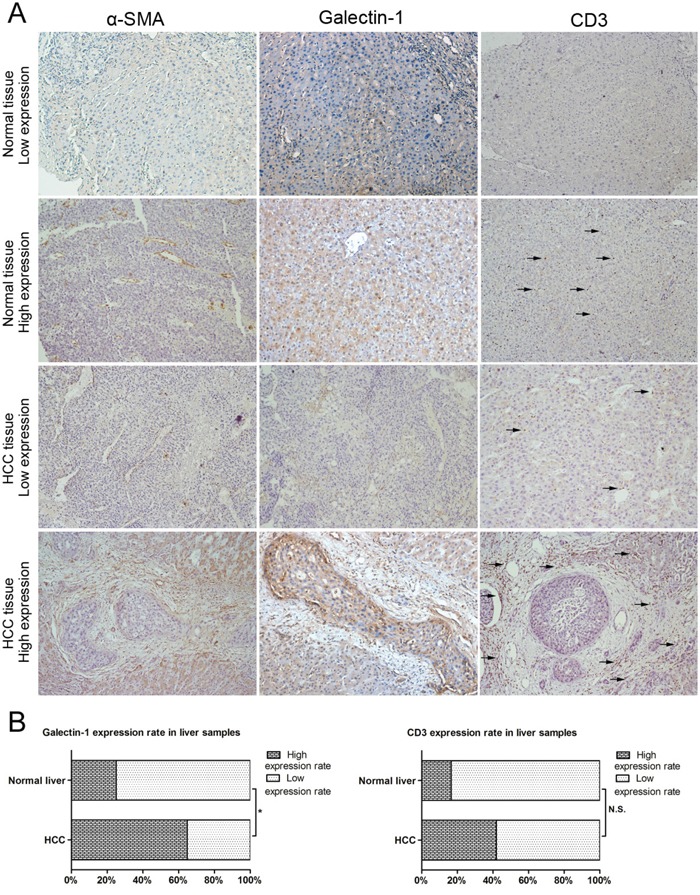
Expression of galectin-1, α-SMA and CD3 in liver samples **A.** Galectin-1, α-SMA, and CD3 levels in HCC liver samples (n = 162) and normal liver samples (n = 12) (paraffin-embedded specimens) measured by IHC, scored and grouped into low expression and high expression groups. **B.** Galectin-1 and CD3 levels in HCC and normal liver samples analysed using the χ^2^ test. Magnification: ×100. N.S. for P > 0.05; *P < 0.05.

Moreover, we tested 162 HCC liver samples and 12 normal liver samples (paraffin-embedded specimens) for the expression of galectin-1, α-SMA, and CD3 using IHC. All of the samples were scored and grouped into low or high expression according to the previously described method. Examples of samples with the standard low and high expression of galectin-1, α-SMA, and CD3 can be found in Figure 9A. The IHC results showed that galectin-1 was highly expressed in the stromal cells in HCC while high expression in normal liver was rare. Strong expression of α-SMA and CD3 was found in HCC while the expression in normal liver tissues was limited. The statistical analysis revealed higher galectin-1 expression in HCC than in normal liver samples (Figure 9B).

**Figure 10:**
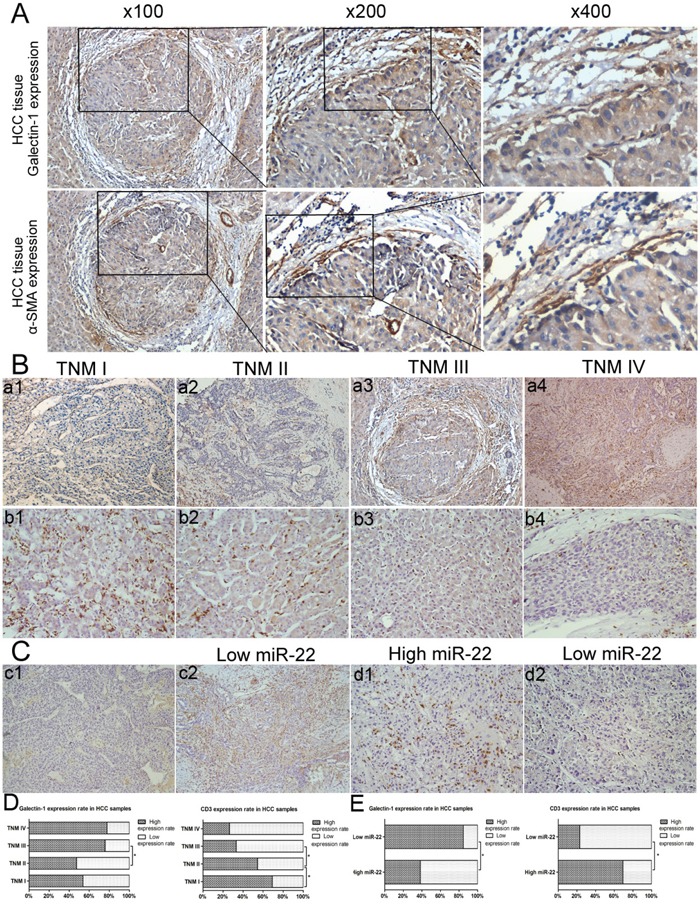
Correlation between miR-22 expression, galectin-1 expression, CD3 expression and the clinicopathological features of HCC **A.** IHC analysis to measure expression of α-SMA and galectin-1 in consecutive sections of HCC samples (n=53). **B-D.** Galectin-1 expression in different TNM stages (TNM I-IV: **A1-A4.**) and CD3 expression in different TNM stages (TNM I-IV: **B1-B4.**) measured by IHC. The correlations between galectin-1 expression, CD3 expression, and TNM stage of patients with HCC were analysed using the χ^2^ test (TNM I, n = 13; TNM II, n = 55; TNM III, n = 58; TNM IV, n = 36). **C-E.** Expression of **C1-C2.** galectin-1 and **D1-D2.** CD3 in Ca-HSCs isolated from HCC patient liver samples, separated into low (n = 13) and high (n = 13) miR-22 expression groups, according to the median miR-22 expression. Magnification: ×200 for CD3 staining (B1-B4, B1-B2), ×100 for galectin-1 staining (a1-a4, c1, c2). *P < 0.05.

We also performed co-expression analyses of α-SMA and galectin-1 on 53 consecutive section samples, which were randomly selected from 162 HCC paraffin-embedded sections (Figure 10A). The co-expression level was scored by evaluating the proportion of co-expression stroma region (which was positive for both galectin-1 and α-SMA) over the total proportion of galectin-1-positive stroma region (positive for galectin-1). The IHC analysis revealed that galectin-1 was highly expressed in stromal cells in HCC, and most of them (mean percentage: 87.34±5.36) were co-expressed with α-SMA. The further substantiates the correlation between galectin-1 expression in stromal cells in HCC and HSC-derived galectin-1 expression. Thus, in assessing the HSC-derived galectin-1 expression level in HCC by IHC, we only included the positive staining in stromal cells for galectin-1 scoring.

The correlations between IHC results for galectin-1 and CD3 expression and the clinicopathological features of patients with HCC were analysed using the *χ*^2^ test. As previously mentioned, only the positive staining in stromal cells was included for galectin-1 scoring, and the numbers, but not the intensity, of positively-stained cells were included in the CD3 scoring. As shown in Table 1, some clinicopathological features of HCC (including tumour size, differentiation, TNM stage and distant metastasis) were closely correlated with high galectin-1 expression and low CD3 expression in HSCs. However, we observed no correlation between galectin-1 or CD3 expression and the other parameters, including age, sex and AFP. As shown in Figure 10B and 10D, the positive expression rate of galectin-1 in stromal cells increased with increasing TNM stages, whereas the expression of CD3 exhibited the opposite trend. Moreover, correlation analyses (Table 2) indicated that CD3 expression correlated negatively with galectin-1 expression in HCC. Taken together, these results indicate that the high expression of galectin-1 in HSCs is correlated negatively with the reduced number of infiltrating CD3^+^ T cells and tumour development in HCC.

**Table 1:** Correlation between HSC-derived galectin-1 expression, CD3 expression and the clinicopathological features of patients with HCC

Variable	All cases	Galectin-1		χ^2^	*P* value	CD3		*χ*^2^	*P* value
		Low expression	High expression			Low expression	High expression		
Age (years)									
≤ 50	67	23 (34.3%)	44 (65.7%)	0.037	0.848	37 (55.2%)	30 (44.8%)	0.368	0.544
> 50	95	34 (35.8%)	61 (64.2%)			57 (60.0%)	38 (40.0%)		
Sex									
Male	127	42 (33.1%)	85 (66.9%)	1.152	0.283	75 (59.1%)	52 (40.9%)	0.256	0.613
Female	35	15 (42.9%)	20 (57.1%)			19 (54.3%)	16 (45.7%)		
AFP									
< 200 ng/dL	106	37 (34.9%)	69 (65.1%)	0.011	0.918	65 (61.3%)	41 (38.7%)	1.368	0.242
> 200 ng/dL	56	20 (37.5%)	36 (62.5%)			29 (51.8%)	27 (48.2%)		
Tumour size (cm)									
≤ 5	68	41 (60.3%)	27 (39.7%)	7.655	0.006	32 (47.1%)	36 (52.9%)	5.786	0.016
> 5	94	36 (38.3%)	58 (61.7%)			62 (66.0%)	32 (34.0%)		
Differentiation									
Well	21	8 (38.1%)	13 (61.9%)	8.688	0.034	10 (47.6%)	11 (52.4%)	11.343	0.010
Moderate	87	30 (34.5%)	57 (65.5%)			46 (52.9%)	41 (47.1%)		
Poor	45	12 (26.7%)	33 (73.3%)			35 (77.8%)	10 (22.2%)		
Undifferentiated	9	7 (77.8%)	2 (22.2%)			3 (33.3%)	6 (66.7%)		
TNM stage									
I	13	6 (46.2%)	7 (53.8%)	13.864	0.003	4 (30.8%)	9 (69.2%)	12.729	0.005
II	55	29 (52.7%)	26 (47.3%)			25 (45.5%)	30 (54.5%)		
III	58	14 (24.1%)	44 (75.9%)			39 (67.2%)	19 (32.7%)		
IV	36	8 (22.2%)	28 (77.8%)			26 (72.2%)	10 (27.7%)		
Distant metastasis									
M0	128	51 (39.8%)	77 (60.2%)	5.804	0.016	68 (53.1%)	60 (46.9%)	6.011	0.014
M1	34	6 (17.6%)	28 (82.4%)			26 (76.5%)	8 (23.5%)		

**Table 2:** Correlation between HSC-derived galectin-1 expression and CD3 expression in patients with HCC

			CD3		Total	*χ*^2^	*P* value
			High expression	Low expression			
Galectin-1		High expression	35	70	105		
		Low expression	33	24	57	9.151	0.002
	Total		68	94	162		

### MiR-22 expression in HSCs is correlated with galectin-1 and CD3 expression in HCC

We also performed IHC analyses on HCC samples to verify the role of miR-22 in HCC. Primary Ca-HSCs were isolated from HCC patient liver samples (n=26) and separated into two groups (low and high miR-22 expression) according to the median HSC-derived miR-22 expression result. We tested both groups for galectin-1 and CD3 expression using IHC (Figure 10C). The correlations among miR-22, galectin-1, and CD3 expression in HCC samples were subsequently analysed using the *χ*^2^ test (Fisher's exact test). As shown in Figure 10E, high miR-22 expression was closely correlated with low galectin-1 expression and high CD3 expression in HCC samples. Taken together, the above-described results suggest that the high expression of galectin-1 in HSCs can promote local immune privilege (most likely by reducing the number of CD3^+^ T cells and skewing the Th1/Th2 cytokine balance) and tumour development in HCC and that, these effects can be inhibited by miR-22.

### High expression of galectin-1 and low expression of CD3 correlate with shorter survival of patients with HCC

**Figure 11:**
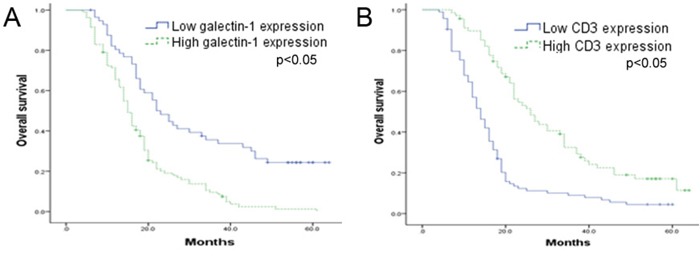
Kaplan-Meier curve of overall survival based on galectin-1 and CD3 expression in 162 HCC patients HCC patients were grouped into high (n = 105) and low (n = 57) galectin-1 expression groups, or high (n = 68) and low (n = 94) CD3 expression groups. The correlation between **A.** galectin-1 or **B.** CD3 expression and survival was analysed using the Kaplan-Meier method. The P-values were determined using the log-rank test.

We also examined the prognostic value of HSC-derived galectin-1 and CD3 expression. As shown in Figure 11, high HSC-derived galectin-1 expression and low CD3 expression correlated with shorter survival time. Furthermore, all of the parameters showing significance in the univariate analysis were tested in a Cox multivariate proportional hazards regression analysis. As shown in Table 3, HSC-derived galectin-1 and CD3 expression were found to be independent prognostic indicators for the survival of patients with HCC.

**Table 3:** Univariate and multivariate analyses of prognostic parameters and galectin-1 and CD3 expression in patients with HCC

Variable	Univariate		Multivariate	
	HR^a^ (95% CI^b^)	*P* value	HR^a^ (95% CI^b^)	*P* value
Age (years) (> 50 vs. ≤ 50)	1.171 (0.837-1.640)	0.357		
Sex (female vs. male)	1.010 (0.674-1.513)	0.963		
AFP (> 200 ng/dL vs. < 200 ng/dL)	1.038 (0.730-1.477)	0.835		
Tumour size (cm) (> 5 vs. ≤ 5)	1.467 (1.043-2.063)	0.028	0.819 (0.551-1.217)	0.323
Differentiation	1.989 (1.259-3.143)	0.003	1.725 (1.072-2.777)	0.025
(Moderate/Well vs. Undifferentiated/Poor)				
Stage (III/IV vs. I/II)	1.907 (1.353-2.688)	< 0.001	1.309 (0.903-1.898)	0.155
Distant metastasis (M1 vs. M0)	2.000 (1.341-2.981)	0.001	1.628 (1.057-2.506)	0.027
Galectin-1 intensity (High vs. Low)	2.460 (1.687-3.587)	< 0.001	2.218 (1.398-3.521)	0.001
CD3 intensity (High vs. Low)	0.390 (0.275-0.554)	< 0.001	0.435 (0.301-0.628)	< 0.001

Bold items indicate *P* < 0.05.

a HR: hazard ratio.

b CI: confidence interval.

## DISCUSSION

Increasing evidence points to the importance of the microenvironment in tumour development [23]. More specifically, a strong immune microenvironment can control tumour progression, while a state of immune tolerance might promote cancer [23]. In the liver, HSCs are known modulators of the tumour cell microenvironment [2]. However, the underlying mechanisms of HSCs' role in HCC are not fully understood. In this study, our results indicated that HSCs could promote tumour immune privilege and tumour progression through galectin-1 expression, while this function can be suppressed by miR-22.

HSCs are fat-storing pericytes of the liver, which store vitamin A, impact sinusoidal blood flow, and maintain the hepatic architecture (extracellular matrix synthesis) [5]. Under inflammatory conditions, quiescent HSCs become activated and undergo changes in morphology and biological function [21]. With stimulation from the surrounding environment, activated HSCs can produce large amounts of functional molecules, such as growth factor and cytokines, which in turn affect the environment [2]. Several years ago, Qian *et al*. reported that HSCs could inhibit T cells, mainly by inducing apoptosis [5]. Recently, several immune-related molecules, such as B7-H1, have been reported to partially account for HSCs' immunosuppressive effect [24, 25]. However, inhibition of any one of these molecules alone cannot completely remove the immunosuppressive function of HSCs [5, 24, 25], suggesting that a network of several molecules is responsible for this effect. Galectin-1is immunosuppressive since it induces apoptosis of activated T cells [6, 7], which is similar to the immunomodulatory mechanism of HSCs. In this study, we found that galectin-1 is an important functional molecule that activated HSC expression and release. Our results showed that regulating galectin-1 expression levels in HSCs could significantly affect their ability to promote apoptosis of T cells and skew the balance of Th cytokine. These results suggest that galectin-1 contributes to HSCs' immunomodulatory function. Previous studies have detected galectin-1 expression in rat HSCs [16, 17]; however, galectin-1 expression in human HSCs and the relationship between galectin-1 and the immunomodulatory functions of HSCs have not yet been reported.

Much evidence has indicated that HSCs can induce the development of HCC by promoting tumour immune privilege [1, 2]. In this study, our results suggested that galectin-1 might be involved in this process. Many studies have reported the immunosuppressive properties of galectin-1, which might correlate with the development of tumours [6, 7]. In HCC, galectin-1 is considered an effective marker for predicting poor prognosis [12, 13]. Although galectin-1 was detected in tumour hepatocytes [14, 15], higher galectin-1 expression in stromal cells than in hepatocytes suggested that stromal cells might regulate galectin-1 expression in HCC. In this study, Ca-HSCs exhibited higher galectin-1 expression and more powerful immunosuppression than N-HSCs. Moreover, CD3 expression (representative of infiltrating T cell numbers) and HSC-derived galectin-1 expression were negatively correlated. Furthermore, HSC-derived galectin-1 expression correlated negatively with prognosis of HCC patients, while CD3 expression correlated positively with patient prognosis. Although the question of which source of galectin-1 (tumour hepatocytes or HSCs) contributes more to the progression of HCC has not been answered definitively, our results suggested that HSC-derived galectin-1 promotes HCC progression at least in part by affecting T cells. However, in addition to TNM stage, tumour differentiation also correlated with HSC-derived galectin-1 and CD3 expression. Moreover, both HSC-derived galectin-1 and CD3 expression levels were found to be independent prognostic indicators for HCC patient survival. Taken together, these results suggest that a) HSC-derived galectin-1 can promote HCC by other mechanisms that are irrelevant to immune privilege, and b) not all factors that influence the number of T cells are affected by galectin-1 expression. Nevertheless, our results indicated that HSC-derived galectin-1 correlated with tumor immune privilege and HCC progression.

Our results suggested that dysregulation of miR-22 expression in HSCs can result in overexpression of HSC-derived galectin-1. Recent studies have reported that miR-22 dysregulation in many types of cancer [26–29]. In HCC, miR-22 has been shown to be a downregulated tumour suppressor; however, the mechanisms of its anti-tumour effects remain unclear [19]. In this study, our results indicated a direct, negative regulatory relationship between galectin-1 and miR-22 expression in HSCs, as well as a role for miR-22 in the HSC-derived immunomodulation. In addition, miR-22 overexpression suppressed HSC-derived immunosuppression; yet, this could be almost completely reversed by galectin-1 overexpression. This indicates that galectin-1 can counteract the miR-22-induced inhibition of HSC-derived immunosuppression. We observed higher HSC-derived miR-22 expression and lower HSC-derived galectin-1 expression in HCC samples than in normal liver tissues. Moreover, in HCC samples, the correlation between miR-22 and galectin-1 expression and CD3 expression suggested a correlation between miR-22 and galectin-1 and infiltrating T cells. Taken together, our results suggested that miR-22 regulates HSC-derived galectin-1 expression.

Nevertheless, there is also the question of why miRNA-22 expression is deregulated in Ca-HSCs. Many studies have demonstrated that exposure of HSC to conditioned media derived from HCC tumour cells results in HSC activation, migration and expression of pro-angiogenic factors [2, 30, 31]. Such observations suggest that HCC tumour cells secrete functional factors that modify the biology of HSCs. In addition, a recent study by Jinpiao Lin *et al*. found that the tumor-suppressor protein p53 could regulate the expression of miRNA-22 in rheumatoid arthritis [32]. Taken together, we speculate that the dysregulation of p53 expression brought by the interaction between HCC tumour cells and HSC might lead to downregulation of miRNA-22 expression and galectin-1 overexpression in Ca-HSCs. However, further study is still needed to test this hypothesis.

In conclusion, we demonstrated that (a) human activated HSCs can express and release galectin-1, (b) HSC-derived galectin-1 promotes HSC-induced T cell apoptosis and Th1/Th2 cytokine balance skewing, (c) HSC-derived galectin-1 promotes HCC progression by improving immune privilege, and (d) all aforementioned effects can be inhibited by miR-22. Overall, our findings suggest that HSC-derived galectin-1 and miR-22 could be used as prognostic markers and therapeutic targets for HCC.

## MATERIALS AND METHODS

### Patients and tissues

Liver tissue samples were collected from patients (162 with HCC and 12 with benign liver diseases) who underwent hepatectomy at the Department of Hepatobiliary Surgery (Southwest Hospital, Third Military Medical University, China) between 2009 and 2011 according to protocols approved by the Medical Ethics Committee of the Southwest Hospital and the First Affiliated Hospital of the Third Military Medical University. Normal liver tissue samples were obtained from partial hepatectomies for benign liver diseases (hepatic cavernous haemangioma without cirrhosis) and were used as control samples. All patients provided written informed consent for the use of their liver tissue for clinical research. No patients received radiotherapy or chemotherapy prior to the hepatectomy. The survivors were followed for five years. The tumours were pathologically graded according to the World Health Organization classification system, and pathological staging after hepatectomy was performed according to the 2002 tumour-node-metastasis (TNM) classification of malignant tumours established by the International Union Against Cancer. The clinicopathological characteristics of the patients with HCC are listed in Table 1. After resection, 26 tumour tissue specimens and 12 normal liver tissues were immediately used for HSC isolation.

### Cells and culture conditions

Human HSCs were isolated from HCC tissues and normal liver tissues using a Nycodenz density gradient centrifugation method. Briefly, liver resections were perfused *ex vivo* with Hanks' balanced salt solution (HBSS; Sigma, USA) containing 1% penicillin-streptomycin solution (Sigma, USA) and then with HBSS containing 0.5 mg/ml collagenase IV (Sigma, USA). The tissue was minced with blunt forceps and scissors and then digested for 20 min with HBSS containing 0.5 mg/ml collagenase IV (Sigma, USA) and 0.1 mg/ml deoxyribonuclease I (Invitrogen, USA). The digested tissue was agitated gently in Dulbecco's modified Eagle's medium (DMEM; Gibco, USA) containing 10% foetal bovine serum (FBS; Gibco, USA) and filtered through 74-μm nylon membranes. The resultant cell suspension was centrifuged three times at 50 × *g* for 5 min and then centrifuged at 500 × *g* for 10 min. The cells were resuspended in DMEM, transferred into HBSS containing 200 mg/ml Nycodenz (Sigma, USA), and then centrifuged at 1400 × *g* for 20 min. The supernatant was then discarded, and the cells were resuspended in DMEM containing 10% FBS and cultured at 37°C in the presence of 5% CO_2_. The HSCs that were isolated using this method were confirmed by morphology assessment and by staining for the HSC-specific marker α-smooth muscle actin (α-SMA). To confirm the expression of galectin-1 in HSCs, the HSCs were seeded onto 96-well plates at a density of 20,000 cells in 200 μl of DMEM per well. After the cells were incubated for 48 h, the cells and cell culture supernatants were collected for subsequent experiments.

Peripheral blood mononuclear cells (PBMCs) from major histocompatibility complex (MHC)-mismatched healthy donors were isolated through density gradient centrifugation with Histopaque 1077 (Sigma, USA) according to the manufacturer's instructions. CD3^+^ T cells were obtained from the PBMCs via fluorescence-activated cell sorting (FACS) with an anti-CD3 antibody conjugated to allophycocyanin (APC) (No. 17-0037, eBioscience, USA). The cells were cultured in RPMI-1640 (Gibco, USA) containing 10% FBS (Gibco, USA), 100 U/ml penicillin G, and 100 μg/ml streptomycin.

### Cell transfection

The following three small hairpin RNA (shRNA) sequences were designed for the knock-down of human galectin-1 expression in HSCs:

sh-1, 5′-CGTCAAGCTGCCAGATGGATACGAA-3′;

sh-2, 5′-GCTGCCAGATGGATACGAATTCAAG-3′; and

sh-3, 5′-ACATGGCAGCTGACGGTGACTTCAA-3′.

A non-targeting scrambled sequence (Scr; 5′-TTCTCCGAACGTGTCACGT-3′) was used as a negative control. The shRNAs (hU6-galectin-1 shRNA, CMV-puromycin) were purchased from SunBio (Shanghai, China). ShRNA transfections were performed using plasmid vectors according to the manufacturer's recommended protocol. Stable cell lines were generated through selection with 2 μg/ml puromycin (SunBio, Shanghai, China) for 15 days. A human galectin-1 overexpression plasmid vector (mCMV-galectin-1, PGK-puromycin) was constructed by SunBio (Shanghai, China). HSCs were transfected using Lipofectamine 2000 (Invitrogen, USA), and stable cell lines were generated through selection with 2 μg/ml puromycin for 15 days. A hsa-miR-22 mimic was used to increase the expression level of miR-22 (Qiagen, Germany), and a negative control mimic (mimic NC) was used as a control (Qiagen, Germany). HSCs were transfected using Lipofectamine 2000 (Invitrogen, USA) according to the manufacturer's recommended protocol.

### Co-culture experiment and T cell apoptosis assay

Isolated CD3^+^ T cells were activated and stimulated with 7.5 μg/ml phytohaemagglutinin (PHA; Invitrogen, USA) for 72 hours and then with 20 U/ml IL-2 (PeproTech, USA) for 24 h. The CD3+ T cells were then harvested and co-cultured with HSCs (subjected to different pre-treatments). To test for pro-apoptotic function of HSCs against T cells, we plated CD3+ T cells (2 × 10^5^/well) and HSCs (isolated from normal liver tissues and pre-cultured for two days or seven days) onto 96-well culture plates at ratios of 20:1, 10:1, and 5:1 in a total volume of 200 μl/well for 48 h. To determine the differences in the immunomodulatory function of HSCs isolated from normal liver tissues (N-HSCs) and HCC tissues (Ca-HSCs), activated CD3+ T cells were co-cultured with N-HSCs or Ca-HSCs for 48 h. To investigate the roles of galectin-1 and miRNA-22 in the immunomodulatory capacity of HSCs, activated CD3+ T cells were co-cultured with N-HSCs with different pre-treatment conditions (such as galectin-1 overexpression and galectin-1 knockdown) for 48 h. After the cells were co-cultured, the CD3+ T cells were harvested and sequentially evaluated through a flow cytometric analysis of apoptosis, and the supernatant was collected for cytokine secretion analysis using an enzyme-linked immunosorbent assay (ELISA).

### Luciferase assay

The 3′-UTR of galectin-1 (82 bp) containing the putative miR-22-binding site (29-35) was synthesized. The gene product was subcloned into a psiCHECK-2 vector (Promega, USA) immediately downstream of the luciferase gene sequence. A psiCHECK-2 construct containing the 3′-UTR of galectin-1 with a mutant seed sequence of miR-22 was also synthesized. All constructs were confirmed by DNA sequencing. HSCs were plated onto 96-well plates and then co-transfected with 100 ng of the constructs with or without the miR-22 expression plasmid. Forty-eight hours after transfection, the level of luciferase activity was detected using a dual-luciferase reporter assay system (Promega, Madison, WI, USA) and normalized against the level of *Renilla* activity.

### Real-time quantitative polymerase chain reaction (RT-qPCR)

The detailed procedures used for total RNA extraction and real-time RT-qPCR were previously described [20]. The primer sequences were synthesized by Invitrogen and are listed in Table 4.

**Table 4:** The primer sets for qPCR

Primer name	Primer sequence
hsa-miR-22-3p RT	5′-GTCGTATCCAGTGCAGGGTCCGAGGTGCACTGGATACGACACAGTTC-3′
hsa-miR-22-3p Fwd	5′-CCAAGCTGCCAGTTGAAGA-3′
U6 RT	5′-GTCGTATCCAGTGCAGGGTCCGAGGTGCACTGGATACGACAAAATATGGAAC-3′
U6 Fwd	5′-GTGCTCGCTTCGGCAGC-3′
U6 Rev	5′-TATCCAGTGCAGGGTCCGA-3′
Galectin-1 Fwd	5′-TCAAACCTGGAGAGTGCCTT-3′
Galectin-1 Rev	5′-CACACCTCTGCAACACTTCC-3′
Tubulin Fwd	5′-TCTACCTCCCTCACTCAGCT-3′
Tubulin Rev	5′-CCAGAGTCAGGGGTGTTCAT-3′

### Western blot (WB) analysis

The galectin-1 protein levels in HSCs were measured by WB analyses with anti-galectin-1 antibody (ab108389, Abcam, USA), and WB was performed as described previously [20]. Each band was normalized against β-tubulin.

### Flow cytometry

After the co-culture experiment, CD3+ T cells were collected and subjected to an evaluation of apoptosis using an Annexin V-FITC Apoptosis Detection Kit (Beyotime Biotechnology, China) and flow cytometry according to the manufacturer's recommended protocol. Briefly, the cells were washed twice with PBS, and annexin V-FITC and PI were added to the binding buffer. The cells were then incubated with the resultant binding buffer mixture for 15 min at room temperature in the dark. The apoptotic cells were then analysed using a BD FACSCalibur flow cytometer (BD Biosciences USA).

### Immunofluorescence assay

The expression level of α-SMA in isolated HSCs was measured by immunofluorescence using an anti-α-SMA antibody purchased from Abcam (USA; ab7817). The expression levels of α-SMA and galectin-1 in HCC and normal freshly-frozen liver specimens were measured by immunofluorescence using anti-α-SMA and anti-galectin-1 antibodies (Abcam, USA, ab108389). The experimental procedure was performed as previously described [20]. The cell nuclei were stained with DAPI.

### Immunohistochemical (IHC) assay

The expression levels of galectin-1, CD3, and α-SMA in formalin-fixed paraffin-embedded samples were determined through IHC with primary antibodies purchased from Abcam (USA; ab108389, ab16669, and ab7817). The liver tissue samples were stained as previously described [20]. The IHC scoring was quantified into four grades according to the proportion and intensity of the stained cells: 0 = no expression; 1+ = weak expression; 2+ = moderate expression, and 3+ = strong expression. For the statistical analysis, the scores were grouped into two categories: low expression (0 and 1+) and high expression (2+ and 3+). To assess the HSC-derived galectin-1 expression level, only the positive staining in stromal cells was included for galectin-1 scoring. To evaluate the number of infiltrating T cells, only the proportion of the positively stained cells was included in the count used for the scoring of CD3 expression. The expression levels of α-SMA and galectin-1 in isolated HSCs were investigated through IHC using a similar procedure without deparaffinization.

### ELISA

To determine whether HSCs can release galectin-1, the cell culture supernatants were analysed using standard ELISA kits (Boster, China). ELISA was also performed to detect the levels of secreted interferon (IFN-γ) and IL-10 in the cell culture supernatants. All assays were performed using four separate samples (two replicates for each sample). The ELISA plates were read at OD450 using a microplate ELISA reader.

### Statistical analysis

The statistical analysis was performed using SPSS v19.0 software. The data from representative experiments are presented as the means ± standard deviation (SD). Comparative analyses were conducted using one-way ANOVA tests and independent t tests, followed by Tukey's post hoc tests. The correlations among miRNA-22 expression, galectin-1 expression, CD3 expression and clinicopathological parameters were evaluated using the *χ*^2^ test or Fisher's exact test. The Kaplan-Meier method and log-rank test were used to perform the univariate survival analysis. Multivariate analysis was performed using the Cox proportional hazards regression model. A *P* value of less than 0.05 was considered significant.
